# Pancreas divisum unmasked: a case report

**DOI:** 10.11604/pamj.2024.49.48.45004

**Published:** 2024-10-22

**Authors:** Imane Zouaki, Mariama Jarti, Asmaa Sadik, Oussama Nacir, Fatimezzahra Lairani, Adil Aiterrami, Sofia Oubaha, Zouhour Samlani, Khadija Krati

**Affiliations:** 1 Department of Hepato-Gastroenterology, University Hospital of Mohammed VI, Marrakech, Morocco

**Keywords:** Pancreas divisum, recurrent acute pancreatitis, magnetic resonance cholangiopancreatography, Morocco, case report

## Abstract

Pancreas divisum occurs during development when the ventral and dorsal buds of the pancreas do not fuse. It is the most common congenital anomaly of the pancreas. Patients with this condition are usually asymptomatic, but almost 25% of these patients experience recurrent pancreatitis, which may progress to chronic pancreatitis. This is a case of a 16-year-old male with a significant history of recurrent pancreatitis. The patient underwent a computed tomography scan which revealed pancreatitis. Further magnetic resonance cholangiopancreatography supported the diagnosis of pancreatic divisum. This case highlights the importance of considering even rare etiologies such as pancreas divisum in unexplained cases of recurrent pancreatitis. After ruling out the obvious etiologies (gallstones, alcohol intake, metabolic disorders), it is recommended to demand a magnetic resonance cholangiopancreatography (MRCP) to better analyze the anatomy of the biliary and the pancreatic ductal systems. Early confirmation of the underlying etiology and aggravating factors can improve patient outcomes and prevent the recurrence of pancreatitis.

## Introduction

Pancreas divisum (PD) is a congenital malformation of the pancreatic ductal system which is characterized by the failure of fusion of the ventral and dorsal pancreatic ducts in the seventh week of foetal development [1]. It´s a rare condition found in about 0.5-10% of the population with a lower reported rate of 1-2% in the Asian and African populations [2]. Most cases of PD are asymptomatic (95%) [2]. However, some may be revealed by recurrent acute pancreatitis (RAP), pancreatic-type pain or even chronic pancreatitis [2]. Treatment options for symptomatic PD include endoscopic and surgical procedures. Endoscopic sphincterotomy of the minor papilla is the treatment of choice and may resolve up to 75% of the pain cases, however, its complications are not rare and can be dangerous [3,4]. Moreover, the cannulation of the minor papilla can prove difficult and fail in up to 20% of the cases [3,4]. In these cases or in the presence of complications such as major pancreatic change or fibrosis, surgery is the best option [4].

Until this day, there has been no consensus on the management of PD, and because of its rarity, it is difficult to recruit enough patients for prospective randomized trials. In their absence, case reports and series are still the main source of information on this disease. To our knowledge, cases of PD in Morocco are rare and only one case has been reported so far [5]. Here, we present a case of acute-on-chronic epigastric pain secondary to pancreatitis due to PD. During hospitalization, the patient was treated medically for acute pancreatitis and later discharged with recommendations for corrective surgery due to the recurrence of the symptomatology, to the multiple stenosis in the pancreatic ducts, and to the presence of gallstones.

## Patient and observation

**Patient information:** a 16-year-old boy, with a history of asthma, was diagnosed with acute pancreatitis of unknown etiology three times and was treated with symptomatic treatment. The patient was then referred to our department for an etiological assessment of recurrent acute pancreatitis.

**Clinical findings:** at admission, the patient was asymptomatic but reported an alteration of general status marked by anorexia and profound weight loss. Physical examination noted a mild epigastric tenderness.

**Timeline of events:** the patient had his third episode of pancreatitis a month prior to his admission to our institution. He brought an abdominal magnetic resonance imaging (MRI) revealing acute pancreatitis related to a pancreatobiliary malformation (pancreas divisum). Upon his admission, an MRCP and a subsequent 3D reconstruction were done confirming the diagnosis of PD and revealing multiple complications.

**Figure 1:**
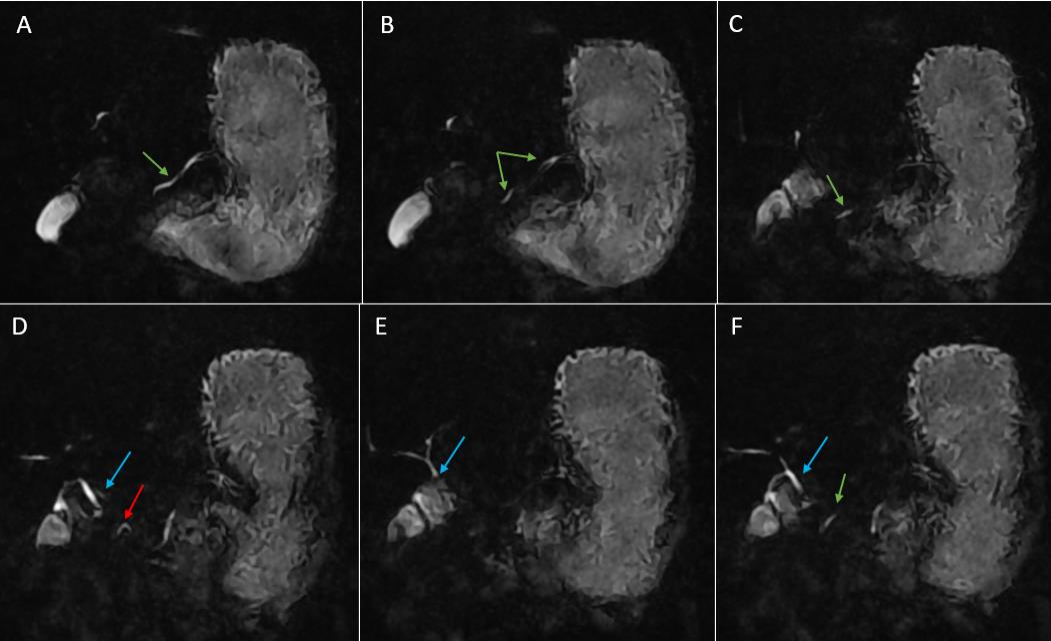
A,B,C,D,E,F) magnetic resonance cholangiopancreatography sequences objectifying the main pancreatic duct (green arrow) of normal diameter crossing a part of the ventral pancreatic duct (red arrow); D) that was not explored in total (multiples stenosis) and the common bile duct (blue arrow) with a serpiginous gallbladder and multiple cholesterol microlithiasis

**Figure 2:**
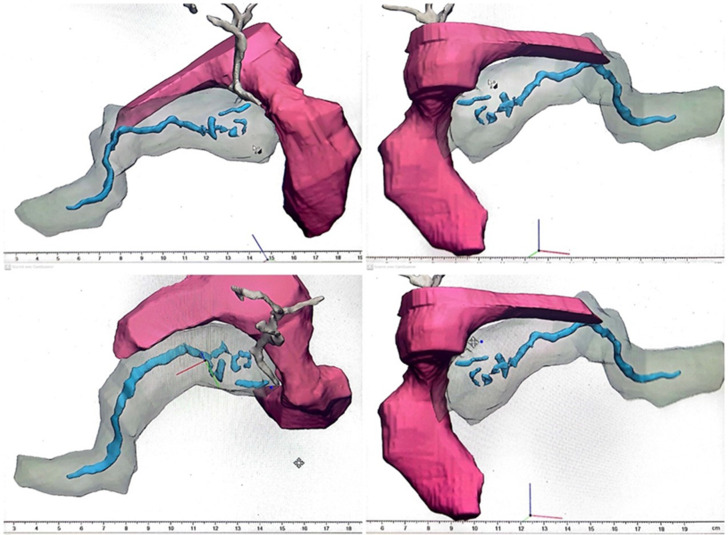
three-dimensional reconstruction using magnetic resonance cholangiopancreatography sequences of our patient showing multiple stenosis in both the main and the accessory pancreatic ducts

**Diagnostic assessment:** upon his admission, laboratory findings noted a normal white blood cells count (5510/ml) (normal: 4000-10000/ml), and a normal C-reactive protein of 0.3 mg/l (0-5mg/l) and normal lipase of 5.4 IU/l (normal: <60 IU/L). His hepatic and renal workups were within normal ranges as well. After ruling out gallstones and other metabolic causes of acute pancreatitis. An abdominal MRI revealed acute pancreatitis classified as Balthazar stage E, which may be related to a pancreatobiliary malformation: pancreas divisum, with a narrowing of the lumen of the second portion of the duodenum and a peritoneal effusion of medium abundance. Magnetic resonance cholangiopancreatography objectified the main pancreatic duct of normal diameter crossing a part of the ventral pancreatic duct that was not explored in total (multiples stenosis) and the common bile duct (crossing duct sign) thus confirming the diagnosis of pancreas divisum with a serpiginous gallbladder and multiple cholesterol microlithiasis (Figure 1). A 3D reconstruction was done using MRCP sequences objectifying multiple stenosis in the ventral and medial part of the main pancreatic duct and in the accessory duct (Figure 2).

**Therapeutic intervention:** the patient was then advised not to consume more than 20 g of fat a day and to undergo surgical correction as per current gastrointestinal recommendations.

**Follow-up and outcomes:** the parents of the patient preferred to seek surgical treatment outside of our institution and were unfortunately lost to follow-up.

**Patient perspective:** the patient and his guardians were satisfied with finally understanding the cause of his recurrent acute pancreatitis and hopeful to be able to do the surgery and obtain a full recovery.

**Informed consent:** it was obtained from the patient and his guardians for the use of his imaging and clinical data for publication and academic research with respect of anonymity.

## Discussion

Pancreas divisum is the most common congenital anomaly of the pancreatic ductal system. Still, it´s a rare condition found in about 0.5-10% of the Western population with an even lower reported rate of 1-2% in the Asian and African populations [2]. To the best of our knowledge, our case is the second reported case in our country (Morocco) and the first case in our region (Marrakech). The other reported case was from Casablanca and was recently reported in 2023 also of a PD revealed by RAP [5].

There are three subtypes of pancreas divisum: the most common one is type 1 or “classic/complete PD” where the ventral duct of Santorini is drained via the major papilla and the dorsal duct of Wirsung is drained via the minor papilla with no communication between the two, type 2 or “dorsal duct PD” where the ventral duct is absent and type 3 or “incomplete PD” which is similar to type 1 except for a small communication between the two ducts [2]. Pancreas divisum (PD) is usually asymptomatic and only 5% of patients present with either RAP, chronic pancreatitis, or chronic abdominal "pancreatic-type" pain [2]. The clinical relevance of PD has always been debatable; the predominant hypothesis incriminates the increase of the intraductal pressure caused by the drainage of the dorsal duct through the minor papilla impairing the pancreatic secretion and causing inflammation [2]. While others consider PD as a cofactor to an underlying genetic mutation as that of serine protease inhibitor Kazal type 1 gene (SPINK1), cystic fibrosis transmembrane conductance regulator gene (CFTR), chymotrypsin C gene (CTRC), and others [2,6]. However, the reasons as to why some patients develop symptoms and others do not are not yet clear. Unfortunately, our patient was in the 5% of the symptomatic cases and presented with RAP.

The diagnosis in our case was suggested by an abdominal MRI and confirmed by an MRCP. The key imaging feature indicating PD is the crossing duct sign; the appearance of the dorsal pancreatic duct running across the intrapancreatic bile duct to join the minor papilla [7]. This is usually seen in both endoscopic retrograde cholangiopancreatography (ERCP) and magnetic resonance cholangiopancreatography (MRCP). Sometimes, on MRCP the duct of the ventral system may not be or barely visible due to its small caliber as in our case.

For the diagnosis of PD, ERCP was considered the gold standard [8]. However, it is an invasive procedure with multiple potential complications such as pancreatitis, perforation, and haemorrhage [8]. With the advent of non-invasive imaging modalities such as MRCP, multi-detector computed tomography (MDCT), and endoscopic ultrasound (EUS), ERCP has now more of a therapeutic role. MRCP is a fast, non-invasive method of visualization of the pancreatobiliary tree and has the same accuracy compared to ERCP in the detection of congenital pancreaticobiliary malformations, including PD [2,8]. Secretin-enhanced MRCP (S-MRCP) was developed specifically for the visualization of the pancreatic ducts. The injection of secretion increases pancreatic exocrine secretions which cause dilation of the pancreatic ducts offering a better visualization of the ductal variations and disorders thus improving the diagnostic performance of MRCP [2]. Finally, EUS is another minimally invasive test which has the advantage of evaluating the details of the pancreaticobiliary ductal system without injecting contrast into these ducts. A systematic review and meta-analysis compared the diagnostic accuracies of MRCP, S-MRCP, and EUS in the detection of PD and concluded that S-MRCP was more reliable than MRCP and EUS. The sensitivities for MRCP, EUS, and S-MRCP in this review were 59%, 83%, and 85%, respectively, and all three imaging techniques had specificities above 97% [9].

In addition to PD, our patient presented with gallstones without cholelithiasis or dilation of the biliary ducts. This was objectified by the last MRCP while imaging during the previous pancreatitis episodes were all negative for gallstones. In a study by Izzo *et al*. seven other cases presented with RAP reveal an underlying PD with the presence of thickening of the bile in the gallbladder in imaging [10]. These patients were treated by laparoscopic cholecystectomy with the absence of further episodes of pancreatitis during a follow-up period of 32 months which highly suggests a possible association between PD and the increased precipitation and concentration of bile in the gallbladder thus causing the inadequate drainage in the ductal system [10]. In our case, and since the previous imaging was negative for gallstones or other biliary anomalies while the patient had recurrent episodes of pancreatitis, we have excluded gallstones as the initial cause of his RAP.

Pancreas divisum patients are only treated when they are symptomatic [2]. The principle of treatment would be to decrease the intraductal pressure created to prevent the recurrence of pancreatitis by easing the pancreatic drainage. The treatment of choice for symptomatic PD patients especially those with RAP is ERCP with papillotomy of the minor papilla, with or without stenting/dilation, with reported efficacy of 70 to 90% in the literature [3,4,8]. Surgery is considered in cases of ERCP failure or in cases of CP and/or complications, such as bile duct or main pancreatic duct stenosis. The surgical options include sphincteroplasty or pancreas head resection (Whipple procedure or duodenum preserving pancreas head resection) [4]. In their recent meta-analysis of 1887 PD patients, Hafezi *et al*. showed that surgical treatment might be superior to endoscopic therapies in terms of success rate and complication rate [4]. However, due to the limitations of the study, the authors could not come up with a definitive recommendation and called for non-randomized trials to be conducted to confirm their findings [4]. For the time being, the decision on treatment for symptomatic PD patients is made individually. The choice of either endoscopy or surgery depends on multiple factors patient-related and disease-related.

In our case, our patient was advised to opt for a surgical treatment as per current guidelines due to the presence of multiple stenosis in the pancreatic duct as well as for gallstones and to prevent future episodes of acute pancreatitis.

## Conclusion

In literature, pancreas divisum is still regarded as a rare disease since most publications are case reports. As of the time of this writing, and to the best of our knowledge, there is still no consensus on the diagnostic nor the therapeutic approach to this disease. In the absence of prospective randomised trials, the current evidence is based mainly on reported cases, series, systematic reviews, and meta-analyses. Thus, case reports like ours are important to know the exact epidemiology, the clinical and radiological variations as well as the different diagnostic and therapeutic means of PD patients around the world. We call clinicians to always look for congenital pancreatic duct malformations such as pancreas divisum in acute pancreatitis and especially in the setting of RAP. Once the obvious etiologies (gallstones, alcohol intake, metabolic disorders) are eliminated, it is recommended to demand an MRCP to thoroughly examine the anatomy of the biliary and the pancreatic ductal systems.
